# Supplementary material for: Repeated blood–brain barrier opening with a nine-emitter implantable ultrasound device in combination with carboplatin in recurrent glioblastoma: a phase I/II clinical trial
Source: Nat Commun. 2024 Feb 23;15:1650. doi: 10.1038/s41467-024-45818-7 (PMC10891097; doi:10.1038/s41467-024-45818-7)
Supplement: Supplementary file 1 — Supplementary Information [file 41467_2024_45818_MOESM1_ESM.pdf]

# Repeated blood–brain barrier opening with a nine-emitter implantable ultrasound device in combination with carboplatin in recurrent glioblastoma: a phase I/II clinical trial

Supplementary Information

## Supplementary Table 1. Patient Characteristics.

| Demographics | All Cohorts (N=34) | Cohort C N=15 | Cohort D N=12 |
|---|---|---|---|
| **Sex** | | | |
| Male<br>Female | 19<br>15 | 7<br>8 | 7<br>5 |
| **Age (years)** | | | |
| Mean (SD)<br>Median | 56.4 (10.6)<br>58.0 | 58.4 (10.0)<br>59.0 | 54.3 (12.1)<br>55.0 |
| **Time since initial diagnosis (months) to enrollment** | | | |
| Mean (SD)<br>Median | 21.5 (21.0)<br>13.2 | 22.0 (26.0)<br>11.7 | 17.2 (14.4)<br>11.2 |
| **Recurrence** | | | |
| 1<br>2<br>4 | 32<br>1<br>1 | 15<br>0<br>0 | 12<br>0<br>0 |
| **Tumor Diameter, Max (mm)** | | | |
| Mean (SD) | 30 (13.5) | 30 (14) | 34 (12) |
| **MGMT status** | | | |
| Methylated<br>Unmethylated | 17<br>17 | 10<br>5 | 4<br>8 |
| **IDH wild-type** | | | |
| Yes<br>No | 33<br>1 | 14<br>1 | 12<br>0 |
| **KPS** | | | |
| 100<br>90<br>80<br>70 | 2<br>20<br>7<br>5 | 1<br>9<br>2<br>3 | 1<br>7<br>3<br>1 |
| **Steroid pre-Surgery** | | | |
| Yes<br>No | 10<br>24 | 5<br>10 | 4<br>8 |
| **Steroid pre-Cycle 1** | | | |
| Yes<br>No | 21<br>12 | 9<br>6 | 9<br>3 |
| **Treatment Delivery** | | | |
| Device Implantation | 34[¶] | 15 | 12 |
| Sonication and carboplatin cycles | 90+11[†] | | |
| **Carboplatin Administration** | | | |
| Time sonication to carboplatin [min] | | 63.7 [±9.9] | |
| Time end of carbo – sonication [min] | NA | NA | 13.8 (± 6.5) |

¶ one patient died 7 days after surgery secondary to pulmonary embolism
† 3 patients continued treatment beyond the protocol prescribed 6 cycles at the discretion of the local investigators for a total of 11 additional cycles

**Supplementary Table 2.** Summary of treatment emergent adverse events (TEAEs) for all grade and grade 3 related to the investigational procedure, according to the common terminology criteria for adverse events (CTCAE).

| **System Organ Class/ Preferred Term** | **CTCAE all grade (N=34)** | **CTCAE Grade 3 (N=34)** |
|---|---|---|
| Patients with Any Related TEAEs (Overall) | 33 (97%) | 12 (35%) |
| **Nervous system disorders** | 25 (74%) | 3 (9%) |
| Dizziness | 8 (24%) | |
| Headache | 7 (21%) | |
| Diplopia/ Vision blurred | 6 (17%) | |
| Aphasia | 4 (12%) | |
| Meningocele acquired | 3 (9%) | |
| Paraesthesia | 3 (9%) | |
| Cerebrospinal fluid leakage | 2 (6%) | |
| Dysarthria | 2 (6%) | |
| Presyncope | 2 (6%) | 2 (6%) |
| Seizure | 2 (6%) | |
| Balance disorder | 1 (3%) | |
| Confusional state | 1 (3%) | |
| Hypoaesthesia | 1 (3%) | |
| Lacunar stroke | 1 (3%) | |
| Language disorder | 1 (3%) | |
| Monoparesis | 1 (3%) | |
| **Skin and subcutaneous tissue disorders** | 19 (56%) | 6 (18%) |
| Pain of skin | 19 (56%) | 6 (18%) |
| **General disorders and administration site conditions** | 8 (24%) | 1 (3%) |
| Fatigue/ Asthenia | 6 (18%) | 1 (3%) |
| Implant site pain | 1 (3%) | |
| **Injury, poisoning and procedural complications** | 3 (9%) | |
| Post procedural oedema | 1 (3%) | |
| Scar | 1 (3%) | |
| Subarachnoid haemorrhage | 1 (3%) | |
| **Ear and labyrinth disorders** | 2 (6%) | |
| Vertigo | 2 (6%) | |
| **Infections and infestations** | 2 (6%) | 2 (6%) |
| Wound infection /Postoperative wound infection | 2 (6%) | 2 (6%) |
| **Psychiatric disorders** | 2 (6%) | |
| Anxiety | 1 (3%) | |
| Depression | 1 (3%) | |
