# Supplementary material for: Repeated blood–brain barrier opening with a nine-emitter implantable ultrasound device in combination with carboplatin in recurrent glioblastoma: a phase I/II clinical trial
Source: Nat Commun. 2024 Feb 23;15:1650. doi: 10.1038/s41467-024-45818-7 (PMC10891097; doi:10.1038/s41467-024-45818-7)
Supplement: Supplementary file 2 — Reporting Summary [file 41467_2024_45818_MOESM2_ESM.pdf]

nature portfolio

Corresponding author(s): Alexandre Carpentier

Last updated by author(s): Dec 18, 2023

Reporting Summary

Nature Portfolio wishes to improve the reproducibility of the work that we publish. This form provides structure for consistency and transparency in reporting. For further information on Nature Portfolio policies, see our Editorial Policies and the Editorial Policy Checklist.

Statistics

For all statistical analyses, confirm that the following items are present in the figure legend, table legend, main text, or Methods section.

n/a	Confirmed	
☐	☒	The exact sample size (*n*) for each experimental group/condition, given as a discrete number and unit of measurement
☐	☒	A statement on whether measurements were taken from distinct samples or whether the same sample was measured repeatedly
☐	☒	The statistical test(s) used AND whether they are one- or two-sided<br>*Only common tests should be described solely by name; describe more complex techniques in the Methods section.*
☐	☒	A description of all covariates tested
☐	☒	A description of any assumptions or corrections, such as tests of normality and adjustment for multiple comparisons
☐	☒	A full description of the statistical parameters including central tendency (e.g. means) or other basic estimates (e.g. regression coefficient) AND variation (e.g. standard deviation) or associated estimates of uncertainty (e.g. confidence intervals)
☐	☒	For null hypothesis testing, the test statistic (e.g. *F*, *t*, *r*) with confidence intervals, effect sizes, degrees of freedom and *P* value noted<br>*Give P values as exact values whenever suitable.*
☒	☐	For Bayesian analysis, information on the choice of priors and Markov chain Monte Carlo settings
☒	☐	For hierarchical and complex designs, identification of the appropriate level for tests and full reporting of outcomes
☒	☐	Estimates of effect sizes (e.g. Cohen's *d*, Pearson's *r*), indicating how they were calculated

Our web collection on statistics for biologists contains articles on many of the points above.

Software and code

Policy information about availability of computer code

Data collection	All data collection for this trial was performed by an external CRO (IQVIA) using their proprietary software. MR imaging data was also collected by a separate imaging CRO, Median Technologies.
Data analysis	Matlab and ITK Snap were used for MR image analysis and references are provided in the manuscript for details of the software tools used. A dedicated statistician performed analysis of the clinical data using SAS.

For manuscripts utilizing custom algorithms or software that are central to the research but not yet described in published literature, software must be made available to editors and reviewers. We strongly encourage code deposition in a community repository (e.g. GitHub). See the Nature Portfolio guidelines for submitting code & software for further information.

Data

Policy information about availability of data

All manuscripts must include a data availability statement. This statement should provide the following information, where applicable:

- Accession codes, unique identifiers, or web links for publicly available datasets
- A description of any restrictions on data availability
- For clinical datasets or third party data, please ensure that the statement adheres to our policy

We have included raw data for the figures included in the manuscript. The clinical data is available upon request.

Research involving human participants, their data, or biological material

Policy information about studies with human participants or human data. See also policy information about sex, gender (identity/presentation), and sexual orientation and race, ethnicity and racism.

Reporting on sex and gender	Included in Table 1.
Reporting on race, ethnicity, or other socially relevant groupings	n/a.
Population characteristics	Included in Table 1.
Recruitment	Recruitment was performed by study centers, typically via multidisciplinary tumor board meetings at each institution according to the inclusion/exclusion criteria for the study. Any biases inherent to the study results may be due to the study inclusion criteria: principally that the patients were eligible for additional resection, had good performance status (KPS>=70), and had a tumor diameter of <70 mm. These potential biases are detailed in the "Patient Selection" section of the Methods in the manuscript.
Ethics oversight	The study was performed at four clinical sites in France and two sites in the USA. All patients provided written informed consent in accordance with institutional guidelines. Approval was obtained from the ANSM (French National Health Agency), the FDA (Food and Drug Administration), local institutional review boards (IRB) at Northwestern University and MD Anderson and the Sud Méditerranée V ethics committee (CPP) for sites in France.

Note that full information on the approval of the study protocol must also be provided in the manuscript.

Field-specific reporting

Please select the one below that is the best fit for your research. If you are not sure, read the appropriate sections before making your selection.

- [x] Life sciences
- [ ] Behavioural & social sciences
- [ ] Ecological, evolutionary & environmental sciences

For a reference copy of the document with all sections, see nature.com/documents/nr-reporting-summary-flat.pdf

Life sciences study design

All studies must disclose on these points even when the disclosure is negative.

Sample size	For the Phase 1, a standard 3+3 escalation design was used. For the Phase 2, the number of patients was calculated according to the criteria described in the "Statistics and Reproducibility" Section of the manuscript.
Data exclusions	No data were excluded from the analysis.
Replication	This study was a single-arm Phase 1/2 study and no replication was performed.
Randomization	This was a single-arm Phase 1/2 study and no randomization was performed.
Blinding	Blinding was not relevant to our study as all patients were implanted with the investigational device and treated according to the study protocol.

Reporting for specific materials, systems and methods

We require information from authors about some types of materials, experimental systems and methods used in many studies. Here, indicate whether each material, system or method listed is relevant to your study. If you are not sure if a list item applies to your research, read the appropriate section before selecting a response.

Materials & experimental systems

n/a	Involved in the study	
x		Antibodies
x		Eukaryotic cell lines
x		Palaeontology and archaeology
x		Animals and other organisms
	x	Clinical data
x		Dual use research of concern
x		Plants

Methods

n/a	Involved in the study	
x		ChIP-seq
x		Flow cytometry
	x	MRI-based neuroimaging

Clinical data

Policy information about clinical studies

All manuscripts should comply with the ICMJEguidelines for publication of clinical research and a completedCONSORT checklist must be included with all submissions.

Clinical trial registration	NCT03744026
Study protocol	The study protocol is not publicly available as this was a Phase 1/2 trial and the Sponsor does not wish to make the full study protocol public.
Data collection	Clinical data collection was performed by an external CRO (IQVia) and the study was performed from February 2019-November 2022, as noted in the Methods. Clinical data was collected at six clinical sites in the USA (2) and France (4): Northwestern Memorial Hospital (Chicago), MD Anderson Cancer Center (Houston), Pitie Salpetriere Hospital (Paris), CHU Timone (Marseille), Hospices Civil de Lyon (Lyon), CHU Anger (Angers). All imaging data was collected by a dedicated imaging CRO, Median Technologies.
Outcomes	For the Phase 1 (escalation of the number of active emitters/beams), the main objective was to identify the Maximum Tolerated Dose (MTD) defined as the highest active-beams level at which ≤ 1 DLT occurred in a maximum of 6 patients by cohort. The DLT was defined as any Common Terminology Criteria for Adverse Events (CTCAE), version 5.0, Grade 3 or higher, event at least possibly attributable to the sonication or to the sonication plus carboplatin procedure that occurred within 15 days and that did not respond to optimal medical management (including steroids) within 7 days, including symptomatic intracranial hemorrhage of any grade, seizure of grade 3 or 4 (status epilepticus) regardless of time of resolution or symptomatic stroke of any grade. The MTD was defined as the highest active-beams level at which ≤ 1 DLT occurs in a maximum of 6 patients by cohort. In Cohort A, SC9 at the 3 active beams level was given to 3 patients. In Cohort B, SC9 at the 6 active beams level was given to 3 new patients. In the absence of DLT, SC9 at the 9 active beams level was given to 3 new patients (cohort C). If 0 of the 3 patients experienced a DLT, then the 9 active beams level was determined to be the MTD and those patients of groups C of the phase 1 study were added to the expansion phase 2a patients treated at MTD (groups C, and D), to assess the BBB opening at the maximum number of SonoCloud-9 emitters tolerated. An Independent Data Safety Monitoring Board evaluated safety data and advised continuation of the trial after each cohort of the dose escalation portion.<br><br>In the extension Phase 2a, BBB opening efficacy was evaluated as the percentage of successful ultrasound sessions. A successful ultrasound session was defined by the number of emitters for which the BBB opening was Grade 2 (sub arachnoid and grey matter contrast enhancement) or Grade 3 (sub arachnoid, grey & white matter contrast enhancement) during the first three cycles by comparison of pre- and post LIPU session T1w magnetic resonance imaging (MRI) as defined by in our previous work (28,38).

Plants

Seed stocks	n/a
Novel plant genotypes	n/a
Authentication	n/a

Magnetic resonance imaging

Experimental design

Design type	Resting state
Design specifications	MRIs were obtained at inclusion, post resection surgery, and pre/post sonication for cycles 1-3. Only pre-sonication MRIs were obtained for cycles 4-6.
Behavioral performance measures	n/a

Acquisition

Imaging type(s) | Structual

Field strength | 3.0

Sequence & imaging parameters | T1w spin echo (3D, 256x256, 120 degree flip angle, 0.9375 mm x 0.9375 mm x 0.9 mm slice thickness)
FLAIR (2D, 256 x 240, 3 mm slice thickness, 0.43 mm x 0.43 mm)
SWI/T2* (3D, >3 mm slice thickness)

Area of acquisition | Whole brain

Diffusion MRI ☐ Used ☒ Not used

Preprocessing

Preprocessing software | Bias correction, brain segmentation and non-rigid registration was performed using ITK-snap.

Normalization | Normalization was performed using ITK-snap.

Normalization template | *Describe the template used for normalization/transformation, specifying subject space or group standardized space (e.g. original Talairach, MNI305, ICBM152) OR indicate that the data were not normalized.*

Noise and artifact removal | *Describe your procedure(s) for artifact and structured noise removal, specifying motion parameters, tissue signals and physiological signals (heart rate, respiration).*

Volume censoring | *Define your software and/or method and criteria for volume censoring, and state the extent of such censoring.*

Statistical modeling & inference

Model type and settings | *Specify type (mass univariate, multivariate, RSA, predictive, etc.) and describe essential details of the model at the first and second levels (e.g. fixed, random or mixed effects; drift or auto-correlation).*

Effect(s) tested | *Define precise effect in terms of the task or stimulus conditions instead of psychological concepts and indicate whether ANOVA or factorial designs were used.*

Specify type of analysis: ☐ Whole brain ☒ ROI-based ☐ Both

Anatomical location(s) | Tumor progression was analyzed within an ROI that encompassed the treatment volume with the SonoCloud-9 System.

Statistic type for inference | n/a

(See Eklund et al. 2016)

Correction | n/a

Models & analysis

n/a	Involved in the study
☒	☐ Functional and/or effective connectivity
☒	☐ Graph analysis
☒	☐ Multivariate modeling or predictive analysis
